# Measurement Matrix Optimization and Mismatch Problem Compensation for DLSLA 3-D SAR Cross-Track Reconstruction

**DOI:** 10.3390/s16081333

**Published:** 2016-08-22

**Authors:** Qian Bao, Chenglong Jiang, Yun Lin, Weixian Tan, Zhirui Wang, Wen Hong

**Affiliations:** 1 Science and Technology on Microwave Imaging Laboratory, Institute of Electronics, Chinese Academy of Sciences (IECAS), Beijing 100190, China; chenglong.j@gmail.com (C.J.); ylin@mail.ie.ac.cn (Y.L.); whong@mail.ie.ac.cn (W.H.); 2 University of Chinese Academy of Sciences (UCAS), Beijing 100190, China; 3 College of Information Engineering, Inner Mongolia University of Technology, Hohhot 010051, Inner Mongolia, China; wxtannm@163.com; 4 Department of Electronic Engineering, Tsinghua University, Beijing 100084, China; zhirui1990@126.com

**Keywords:** DLSLA 3-D SAR, measurement matrix optimization, mutual coherence, measurement matrix mismatch, sparse Bayesian inference

## Abstract

With a short linear array configured in the cross-track direction, downward looking sparse linear array three-dimensional synthetic aperture radar (DLSLA 3-D SAR) can obtain the 3-D image of an imaging scene. To improve the cross-track resolution, sparse recovery methods have been investigated in recent years. In the compressive sensing (CS) framework, the reconstruction performance depends on the property of measurement matrix. This paper concerns the technique to optimize the measurement matrix and deal with the mismatch problem of measurement matrix caused by the off-grid scatterers. In the model of cross-track reconstruction, the measurement matrix is mainly affected by the configuration of antenna phase centers (APC), thus, two mutual coherence based criteria are proposed to optimize the configuration of APCs. On the other hand, to compensate the mismatch problem of the measurement matrix, the sparse Bayesian inference based method is introduced into the cross-track reconstruction by jointly estimate the scatterers and the off-grid error. Experiments demonstrate the performance of the proposed APCs’ configuration schemes and the proposed cross-track reconstruction method.

## 1. Introduction

Downward looking sparse linear array three-dimensional synthetic aperture radar (DLSLA 3-D SAR) is a new development of 3-D SAR, with the ability of overcoming layover and shading effects. With linear array antennas distributed along the wing of a rectilinearly moving platform and a downward-looking imaging geometry, DLSLA 3-D SAR can synthesize a 2-D plane array and obtain the 3-D resolution of the imaging scene [1,2,3,4,5,6].

Many algorithms [1,2] for DLSLA 3-D SAR imaging have been proposed based on the assumption that the cross-track antenna phase centers (APC) can form a finite-length uniform linear array. However, the practical cross-track imaging usually suffers from limited resolution and incomplete observation. The former is caused by the length limitation of the array, which is restricted by the platform wingspan. The latter is mainly rendered by the sparse and non-uniform distribution of the APCs, due to the installation restriction, burden of expense and so on [3,4,5]. Considering the spatial sparsity of a 3-D imaging scene, compressive sensing (CS) [7] provides a solution for super-resolution. In the CS theory, measurement matrix plays a crucial role to guarantee the reconstruction performance. The restricted isometry property (RIP) [7] is one of the well-known criteria of measurement matrix, however, it is computationally intractable to explicitly verify [8,9]. For DLSLA 3-D SAR cross-track reconstruction, the measurement matrix is mainly determined by the configuration of the APCs and the grids discretization strategy of the imaging region. When the scatterers are exactly located on the discretized grids, the configuration of the APCs affects the mutual coherence of the measurement matrix, which is an easy-checked substitute for RIP [8,9]. Thus, an optimized configuration of the APCs can improve the performance of sparse recovery. In the case of off-grid, i.e., actual scatterers deviate from the imaging grids, the mismatch problem of measurement matrix will arise [10,11,12,13]. Since a practical scene is always a continuous field with scatterers scarcely on the exact grids, the mismatch problem of measurement matrix is unavoidable and needed to be considered.

Since the sparse and non-uniform configuration of APCs is a sampled subset of a uniform linear array, the measurement matrix can be viewed as a subset of the spatial Fourier basis matrix [5]. We give two metrics to improve the recovery property of the measurement matrix. Based on the worst-case mutual coherence, our first metric optimizes the measurement matrix analytically or numerically. The cyclic difference sets (CDS) [14,15] are used to construct the analytically optimal measurement matrix, while the numerical search method based on the modified Lloyd algorithm [14] is implemented when the analytical construction is not available. On the other hand, the modified average mutual coherence is used as the second metric to optimize the measurement matrix, and we formulate a combination optimization problem with a constraint condition that limits the distribution of the mutual coherence support. The optimized measurement matrix is expected to have a good average reconstruction performance. To solve the mismatch problem of measurement matrix caused by off-grid scatterers, the off-grid sparse Bayesian inference (OGSBI) method [10] is used in this paper for cross-track reconstruction by jointly estimating the scatterers and the grid mismatch error. By applying a hierarchical sparseness prior for the sparse signal and a uniform distribution prior for the mismatch error, the method can obtain a maximum a posteriori (MAP) [16] solution iteratively. With the advantages of no need for regularization parameter and a denser sampling grid, the proposed OGSBI based cross-track reconstruction method can reconstruct scatterers accurately.

The main contribution of this paper lies in concerning the technique to optimize the measurement matrix and deal with the off-grid mismatch for DLSLA 3-D SAR cross-track reconstruction. The proposed methods are novel and useful for DLSLA 3-D SAR imaging, and not much research of this area has been published according to our literature review. The rest of the paper is outlined as follows. Section 2 gives the imaging model and sparse reconstruction conditions for DLSLA 3-D SAR. In Section 3, two measurement matrix optimization metrics are described elaborately. The Bayesian inference based reconstruction algorithm is introduced in Section 4. The extensive experiments are shown to verify the proposed methods in Section 5. Finally, we conclude the paper in Section 6.

## 2. DLSLA 3-D SAR Signal Model and Sparse Reconstruction Conditions Analysis

### 2.1. DLSLA 3-D SAR Imaging Geometry and Signal Model

**Figure 1:**
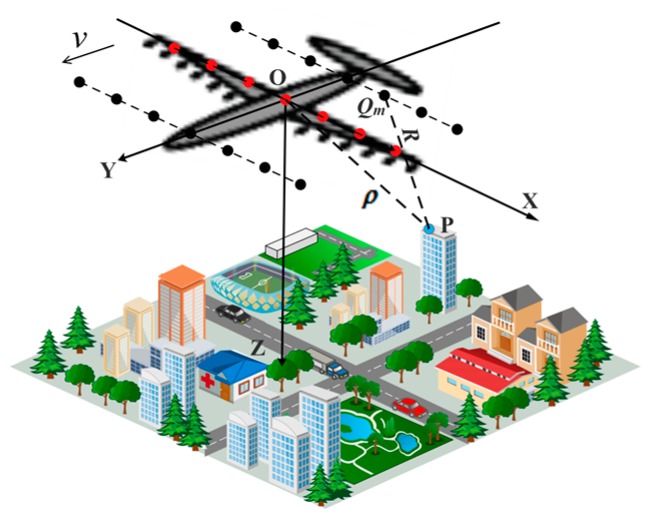
Imaging geometry of DLSLA 3-D SAR.

As shown in Figure 1, DLSLA 3-D SAR observes the nadir imaging scene with a sparse linear array along the wing of the platform (*X*-axis, the cross-track dimension), which moves rectilinearly with a constant velocity *v* along the *Y*-axis, the along-track dimension. *Z*-axis, representing the height dimension, is perpendicularly downward to the *XOY* plane and *O* is the origin. By applying the multiple-input multiple-out (MIMO) array configuration [1], the linear array can achieve co-located transmit-receive APCs according to the equivalent phase center principle [3]. The position of the *m*-th APC is denoted by $Q_{m}$= (*x_m_*,*y_n_*,0) , *m* ∈ [1,*M*], *n* ∈ [1,*N_a_*], where *x_m_* is the cross-track position, *y_n_* is the *n*-th sample in along-track dimension, *M* is the number of APCs in the uniform linear array, and *N_a_* is the along-track sample number. For a point target *P* with coordinates $r_{p}=\left({\hat{x}}_{p},{\hat{y}}_{p},{\hat{z}}_{p}\right),\rho ={\left\|r_{p}\right\|}_{2}$ is its distance from *O*, where ${\left\|●\right\|}_{2}$ denotes the $l_{2}$-norm.

A linear frequency modulated signal with carrier frequency *f_c_*, chirp rate *K_r_* and pulse width *T_p_*, transmitted by the *m*-th APC, is given by:

(1) $$s_{t}(n,m,t)=\text{rect}(\frac{t}{T_{p}})\times \exp(j2\pi f_{c}t+j\pi K_{r}t^{2}),$$

where *t* is the fast time. Signals are transmitted by array antenna elements, reflected by scatterers in the 3-D scene, and then received by all receiver elements. After compensating for the phase error induced by the difference between the APCs and the real transceivers [4], the echo signal can be expressed as:

(2) $$s_{r}(x_{m},y_{n},t)={∭}_{p\in [P]}\sigma (P)\times \text{rect}(\frac{t-t_{d}}{T})\times \exp(-j2\pi f_{c}t_{d}+j\pi K_{r}{\left(t-t_{d}\right)}^{2})dx_{p}\;dy_{p}\;dz_{p},$$

where [*P*] is the support of the 3-D observed scene, *σ*(*P*) represents the radar cross section of target *P*, and *c* is the speed of light. After matched filtering, the frequency domain signal of wave propagation dimension can be written as:

(3) $$s_{r}(x_{m},y_{n},f_{k})={∭}_{p\in [P]}\sigma_{p}\times \exp(-j\frac{4\pi (f_{c}+f_{k})}{c}R(m,n;P))dx_{p}\;dy_{p}\;dz_{p},$$

where *f_k_* ∈ [–*K_r_T*/2,*K_r_T*/2], *k*∈[1,*N_r_*], *N_r_* is the sample number of the wave propagation dimension, and *R*(*m,n*;*P*) = ${\left\|Q_{m}-r_{p}\right\|}_{2}$ denotes the target-to-APC distance.

DLSLA 3-D SAR works in the downward-looking mode, thus the cross-track zero Doppler plane *YOZ* can be chosen as the 2-D focused plane. The 2-D focused images can be obtained by wave-propagation and along-track 2-D dimensional compression. Then, after performing the compensation for the residual video phase terms [4,5], the signal of each wave-propagation and along-track cell is given by:

(4) $$s(x_{m})=\overset{N_{p}}{\underset{p=1}{\Sigma }}\gamma_{p}\times \exp\{j\frac{4\pi x_{m}{\hat{x}}_{p}}{\lambda r_{0}}\},$$

where *γ_p_* is the reflectivity coefficient of the target, *λ* is the nominal radar wavelength, *N_p_* is the total number of point targets, and *r*_0_ is the projection distance of the target-to-APC on the zero-Doppler plane.

Since the radar imaging scene usually contains different kinds of targets, in DLSLA 3-D SAR imaging geometry we mainly consider the scenes that can be modeled by a limited number of strong scattering centers. Thus, the 3-D regions of interested behave typically spatial sparsity, i.e., each wave-propagation and along-track pixel contains only a limited number of dominating scatterers compared with the total cross-track dimension [3,4,5]. Therefore, the cross-track imaging of DLSLA 3-D SAR can be regarded as the problem of sparse signal reconstruction. Suppose that the actual locations of scatterers in cross-track dimension are $\hat{x}=\left[{\hat{x}}_{1},{\hat{x}}_{2},\ldots ,{\hat{x}}_{N_{p}}\right]$, and the cross-track dimension is divided into equal imaging grids $\underset{˜}{x}=[{\underset{˜}{x}}_{1},\;{\underset{˜}{x}}_{2},\cdots ,{\underset{˜}{x}}_{M}]$, where the number of grids is chosen as the number of APCs in the uniform linear array and the grid spacing matches the cross-track nominal resolution. Assuming that the scatterers are exactly on the discretized grids, i.e., $\hat{x}\subset \underset{˜}{x}$, then the matrix expression of each wave-propagation and along-track cell is formulated as:

(5) s=φUγ+n=Rγ+n,

where $U=\left[{\bar{u}}_{1},{\bar{u}}_{2},\ldots ,{\bar{u}}_{M}\right]\subset C^{MxM}$ is the basis matrix (Fourier matrix), the *k*-th column ${\bar{u}}_{k}=[\exp\left(j2\pi \omega_{k}x_{1}\right),\ldots ,exp{\left(j2\pi \omega_{k}x_{M}\right]}^{T}$is viewed as the steering vector, $\omega_{k}=2\pi {\underset{˜}{x}}_{k}/(\lambda r_{0})$, $\varphi \in {\left\{0,1\right\}}^{N_{e}\times M}$ denotes the sampling matrix with elements 1 only at the indices of *N_e_* selected APCs, $R=\varphi U\in C^{N_{e}\times M}$ is the measurement matrix (partial Fourier matrix), $s={\left[s\left(x_{1}\right),\ldots ,s\left(x_{N_{e}}\right)\right]}^{T}$denotes the observation vector obtained from the *N_e_* APCs, ***n*** and ***γ*** = [$\gamma_{1}$,…,$\gamma_{M}$]*^T^* represents the noise vector and the reflectivity coefficients vector, respectively. With *M > N_e_*, the above under-determined equation can be solved with a high probability by sparse signal recovery methods. In the framework of compressive sensing (CS), when there is no prior knowledge of *N_p_* and in the presence of noise, the under-determined equation can be solved by convex $l_{1}$-norm minimization [7]:

(6) $$\underset{\gamma }{\min}\frac{1}{2}{\left\|s-R\gamma \right\|}_{2}^{2}+\tau {\left\|\gamma \right\|}_{1},$$

where *τ* is the regularization parameter. Particularly, if the sparsity prior of the sparse signal is taken into consideration, the solution in Equation (6) is equivalent to a MAP estimation in the Bayesian framework [16]. If $\hat{x}\subset \underset{˜}{x}$ holds, i.e., true scatterers are exactly located at the pre-discretized grids, the above-mentioned grid-based CS can give exact reconstruction, otherwise, off-grid effect will arise [10,11,12,13]. For a practical 3-D SAR imaging scene, the true scatterers are scarcely located on the pre-discretized grids, then the off-grid effect will degrade the reconstruction performance. In Section 4, we will introduce a sparse recovery method based on the Bayesian inference to compensate the off-grid effect.

### 2.2. Sparse Reconstruction Conditions Analysis

In the framework of CS, the measurement matrix plays a crucial role to guarantee the reconstruction performance. The RIP of the measurement matrix provides a sufficient condition [7] for good reconstruction performance, however, it is computationally intractable to explicitly verify [8,9]. Instead, some criteria, e.g., worst-case mutual coherence and average mutual coherence [8,9], are proposed to evaluate the measurement matrix. The mutual coherence, which measures the similarity between different columns of the measurement matrix, is defined as:

(7) $$\mu (u_{i},u_{j})=\frac{\left|\langle u_{i},u_{j}\rangle \right|}{{\left\|u_{i}\right\|}_{2}{\left\|u_{j}\right\|}_{2}},$$

where $u_{i}=\varphi {\bar{u}}_{i}$ is the *i*-th column of the measurement matrix ***R***, and the inner product is $u_{i},u_{j}=u_{i}^{H}u_{j}$. In the model of DLSLA 3-D SAR cross-track reconstruction, substitute the expression of ***u**_i_* and ***u**_j_* into Equation (7), then we get

(8) $$\mu (u_{i},u_{j})=\overset{N_{e}}{\underset{q=1}{\Sigma }}\exp\{j2\pi x_{q}(\omega_{i}-\omega_{j})\}.$$

The worst-case mutual coherence, also known as the maximum mutual coherence, is defined as:

(9) $$\mu (R)=\underset{1\leq i,j\leq M,\;i\neq j}{\max}\mu (u_{i},u_{j})=\underset{1\leq i,j\leq M,\;i\neq j}{\max}\frac{\left|\langle u_{i},u_{j}\rangle \right|}{{\left\|u_{i}\right\|}_{2}{\left\|u_{j}\right\|}_{2}},$$

whose lower bound $\mu {\left(U\right)}_{\text{min}}\geq \sqrt{\frac{M-N_{e}}{N_{e}\left(M-1\right)}}$ is called the Welch bench that is reached for a tight frame [9]. The worst-case mutual coherence measures the dissimilarity between the columns, and it provides a pessimistic measurement for the worst-case reconstruction performance [9,17]. In this condition, the lower bound for the number of observations should satisfy *N_e_* ≥ *C·N_p_·*log(*M/N_p_*), where *C* is a constant that varies with each instance [7].

Another criterion of measurement matrix called the average mutual coherence is defined as:

(10) $$v(R)=\frac{1}{M(M-1)}\max\left|\overset{M}{\underset{i,j=1,j\neq i}{\Sigma }}\langle u_{i},u_{j}\rangle \right|,$$

which gives a measurement of how well the columns spread in the unit hypersphere [8,9]. Many previous studies have investigated the relationship between RIP, worst-case mutual coherence and average mutual coherence, indicating that small worst-case mutual coherence and average mutual coherence can produce high probabilistic guarantees for good reconstruction performance [8,9,17]. For a DLSLA 3-D SAR system, on the afore-mentioned on-grid assumption, the measurement matrix ***R*** is mainly determined by the system parameters and the configuration of the APCs. The former may not be optimized, while the latter is determined by the sampling strategy. Although random sampling strategy has been widely used, there are some methods proposed to optimize the sampling matrix with the aim of decreasing complexity and storage [14,15,18], decreasing the mutual coherence of the measurement matrix, and finally improving the CS reconstruction performance. For DLSLA 3-D SAR cross-track reconstruction, considering the specific expression of the measurement matrix ***R***, we give the following two strategies for sampling APCs from the uniform linear array to form the optimized measurement matrix.

## 3. Measurement Matrix Optimization

### 3.1. Worst-Case Mutual Coherence Based Deterministic Sampling Strategy

For a measurement matrix composed from *N_e_* rows of an *M* × *M* discrete Fourier matrix, random sampling strategy is an obvious method to select the indices of rows, but it needs a large storage and the randomness brings burden on designing complexity in a practical system. Moreover, to find the optimal indices with the lowest worst-case mutual coherence, we need an exhaustive search over $\left(\begin{array}{c} M\\ N_{e} \end{array}\right)$ row indices. As an alternative, a deterministic sampling approach [14,15] has been investigated for the practical implementation. Since the measurement matrix of the DLSLA 3-D SAR cross-track reconstruction model has the similar formulation as a codebook [14], which contains *M* complex vectors in an *N_e_*-dimensional space, the approaches for finding a maximum-Welch-bound-equality codebook [14,19] can be used to find an optimal measurement matrix with a low worst-case mutual coherence. Known from information theory and combinatorial number theory in [14,18], an optimal deterministic sampling strategy based on analytical or numerical method is given as follows.

#### 3.1.1. Analytical Sampling Strategy Based on Cyclic Different Set

**Definition 1.**  ([14]) *An* (*M, N_e_,* *η*) *cyclic difference set (CDS) is defined as a subset* $ID=\left\{id_{1},\ldots ,id_{Ne}\right\}$ *of* $Z_{M}$ *if the N_e_*(*N_e_–*1) *differences* $\left(id_{k}-id_{l}\right)\;mod\;M,\;k\neq l$ *take all possible integers* 1,2,…,*M exactly* *η times, where* $Z_{M}=\left\{0,1,\ldots ,M-1\right\}$ *is the set of integers modulo M. *

**Theorem 1.**  ([14]) *If* **Φ** *is a matrix composed from N_e_ rows of an M* × *M discrete Fourier matrix according to index ID, then* **Φ** *reaches the Welch bench if and only if ID is an (M, N_e_, η) CDS.*

From the earlier analysis we know that measurement matrix ***R*** is composed of *N_e_* rows of the basis matrix (discrete Fourier matrix) ***U***, and the index of the *N_e_* rows is determined by the sampling strategy for cross-track APCs. Thus, according to the Theorem 1 and the related proofs in [14,19], when the sampling index for APCs is an (*M, N_e_,* *η*) CDS, then measurement matrix will reach the minimal worst-case mutual coherence and turns out to guarantee the reconstruction performance in a statistical sense. However, the analytical construction of the deterministic measurement matrix based on CDS is possible only for some certain cases, i.e., not all pairs of (*M*, *N_e_*) exist analytically. The research for CDS or almost difference sets is still under way, and we refer the readers to [20] for more details about the existence of difference sets.

#### 3.1.2. Numerical Sampling Strategy Based on Lloyd Search Algorithm

When analytical construction of the measurement matrix does not exist, a numerical search is needed to find a near-optimal measurement matrix to meet the achievable worst-case mutual coherence. To make the search more efficient, a search method based on modified Lloyd algorithm [14,19] can be employed.

**Figure 2:**
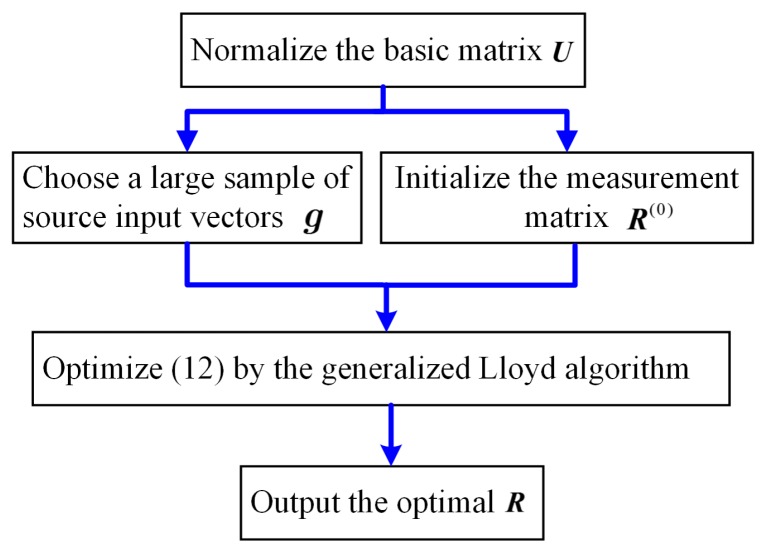
Flowchart of the proposed optimal search method for measurement matrix.

Recall that the columns of the measurement matrix $u_{1}$,…, $u_{M}$ are all *N_e_* × 1 complex vectors constrained on the unit hypersphere $\Omega^{N_{e}}$, the search for the optimal measurement matrix thus can be casted as an equivalent sphere vector quantization (SVQ) problem [21]. In the framework of SVQ problem, the unique distortion metric [14,21] is defined as the projective distance between each source input vector and the vectors to be optimized. To choose a large sample of source input vectors, we firstly normalize each column of the basis matrix, and randomly choose *N_e_* elements from any column to form a source input vector $g\in \Omega^{N_{e}}$. Then the distortion metric between $u_{i}$ and ***g*** is defined as:

(11) $$d(g,\;u_{i})=1-{\left|{u_{i}}^{H}g\right|}^{2},$$

which represents the mutual information between $u_{i}$ and ***g***. The goal of finding the optimal measurement matrix reduces to minimize the average distortion:

(12) $$\begin{array}{l} \underset{R}{\min}\;E_{g}[1-{\left|u_{*}^{H}(g)g\right|}^{2}]\\ \text{s.t. }u_{*}(g)=\arg\underset{u_{i}\in \{u_{1},\cdots ,\;u_{M}\}}{\min}d(g,\;u_{i}), \end{array}$$

where *E**_g_***[●] denotes the ensemble average over all ***g***. To solve the optimization, the generalized Lloyd algorithm [14] is performed to search for the optimal solution. By repeatedly examining the two necessary conditions, i.e., centroid condition and nearest neighbor rule [21], the generalized Lloyd algorithm can converge to a near-optimal solution with a few iterations. We refer the readers to [14,19] for more details about the implementation and the search method is summarized in Figure 2.

For various pairs of *M* and *N_e_*, the numerical method can present a near-optimal measurement matrix with a low worst-case mutual coherence. The search method has low design complexity and the constructed measurement matrix is in a deterministic frame. As will be seen in the experiments, the optimized measurement matrix according to the deterministic sampling strategy, either analytical or numerical, provides a good reconstruction performance.

### 3.2. Modified Average Mutual Coherence Based Sampling Strategy

Different from the worst-case mutual coherence, which gives a pessimistic measurement for the worst-case reconstruction performance, average mutual coherence measures the average reconstruction quality. As defined in (10), most research about the average mutual coherence focuses on the overall distribution of the columns. Considering about the sparse reconstruction requirement for a SAR scene, we propose a modified average mutual coherence with constraint conditions. Firstly, define *C_b_* as the set of the *b* percent of the largest column mutual coherence *μ*($u_{i}$,$u_{j}$), i.e.,:

(13) $$C_{b}=\{\mu (u_{i},u_{j})|\mu (u_{i},u_{j})\geq \varepsilon \},{\underset{\mu (u_{i},u_{j})\in C_{b}}{\Sigma }\mu (u_{i},u_{j})}^{2}=b\underset{i\neq j}{\Sigma }\mu {\left(u_{i},u_{j}\right)}^{2},$$

where *ε* is the threshold. The measure of the mutual coherence support is given by:

(14) $$d_{b}(R)=\underset{\mu (u_{i},u_{j})\in C_{b}}{\max}\left|i-j\right|,$$

which measures the mutual coherence support. Then the metric to find the optimal measurement matrix turns into:

(15) $$\begin{array}{l} \underset{R}{\min}\{\frac{1}{M(M-1)}\underset{i\neq j}{\Sigma }\mu (u_{i},u_{j})\},\\ \text{s.t. }d_{b}(R)/M\leq \beta_{b} \end{array}$$

where 0 < *β_b_* < 1 limits the upper bound of the mutual coherence support. The objective function aims at finding a measurement matrix with a concentrated distribution, and the constraint condition limits the distribution of mutual coherence support, which turns to limit the reconstruction error. When the measure of the support *d_b_*(***R***) is chosen properly, the average mutual coherence will reach a low level. Particularly, when the worst-case mutual coherence of measurement matrix reaches the Welch bound, for ∀ *i*≠*j*, *μ*($u_{i}$,$u_{j}$) = 0 and *d_b_*(***R***) = 0. The proposed mutual coherence metric is expected to give a good predictor of average reconstruction performance and the combination optimization problem in (15) can be solved by algorithms such as simulated annealing algorithm [22].

## 4. Cross-Track Reconstruction Based on Sparse Bayesian Inference

### 4.1. Cross-Track Reconstruction Model with Measurement Matrix Mismatch

Recall that the afore-mentioned CS methods, e.g., the convex $l_{1}$-norm minimization given in (6), are based on the assumption that the scatterers are exactly located on the pre-discretized grids. However, in most SAR imaging scenes, off-grid scatterers cause mismatch problem of the measurement matrix. To solve the problem, the off-grid distance should be taken into account. Following the aforementioned expressions, we have:

(16) $$\Delta x_{k}={\hat{x}}_{k}-{\underset{˜}{x}}_{k},$$

where ${\hat{x}}_{k}$ and ${\underset{˜}{x}}_{k}$ represent the actual cross-track location of the *k*-th scatterer and its nearest grid point, respectively, and Δ*x_k_* is the off-grid distance. Suppose that Δ*x_k_* ∈[–½ς,½ς], where ς is the grid interval of the uniform discretized grids, then the actual steering vector of the measurement matrix can be approximated by the Taylor expansion [10,13]:

(17) $${\overset{⌢}{u}}_{k}\approx u_{k}+\nu_{k}\Delta x_{k},$$

where $v_{k}=u_{k}^{\prime }$ represents the first-order derivative of $u_{k}$. Define ***V*** = [$v_{1},\;\ldots ,v_{M}$], ***Λ*** = *diag*(Δ*x_k_*), *k* = 1,2,…,*Np*, and recall that ***R*** = [***u***_1_, …,***u****_M_*], then the modified matrix-vector expression of (5) is given by:

(18) s=Φ(Λ)γ+n,

where **Φ**(***Λ***) = ***R*** + ***VΛ*** is viewed as the modified measurement matrix. It is worth noting that the afore-mentioned optimization strategies for on-grid measurement matrix ***R*** are still suitable for the modified measurement matrix, since it contains ***R*** and its first-order derivative. Then, to find the support of reflectivity coefficients of the scatterers, we need to jointly estimate ***γ*** and ***Λ***. In the following sub-sections we will briefly introduce the off-grid sparse estimation method in the perspective of Bayesian inference, i.e., OGSBI method [10].

### 4.2. Bayesian Formulation and Sparseness Prior

In the Bayesian framework, the estimates for ***γ*** and ***Λ*** can be cast as the MAP estimation [10]. Considering that the white Gaussian noise is with zero-mean and unknown variance *σ*^2^, the likelihood model can be written as:

(19) $$p(s|\gamma ,\Lambda ,\alpha_{0})={\left(\frac{1}{2\pi \alpha_{0}}\right)}^{N_{e}}\exp(-\frac{\alpha_{0}}{2}{\left\|s-\Phi (\Lambda )\gamma \right\|}^{2}),$$

where *α*_0_ = *σ*^–2^ denotes the inverse of the noise variance. In sparse Bayesian learning, a two-stage hierarchical prior [10,16,23] can be employed to get the sparse prior on ***γ***. Define:

(20) $$\begin{array}{l} p(\gamma |\varepsilon )=\prod_{i=1}^{M}{\left(2\pi \varepsilon_{i}\right)}^{-\frac{1}{2}}\exp(-\frac{{\left|\gamma_{i}\right|}^{2}}{2\varepsilon_{i}})\\ p(\varepsilon |a,b)=\prod_{i=1}^{M}\Gamma (\varepsilon_{i}|a,b), \end{array}$$

where *ε_i_* is the hyper-parameter (inverse-variance) of a Gaussian density function, $\Gamma \left(\varepsilon_{i}|a,b\right)={\left[\Gamma \left(a\right)\right]}^{-1}b^{a}\varepsilon_{i}^{a-1}\text{exp}\left(-b\varepsilon_{i}\right)$ is the gamma distribution with Γ(●) being the Gamma function, *a* > 0 and *b* > 0. Then sparse prior on ***γ*** is obtained by marginalizing over the hyper-parameters ***ε***: p(γ|a,b)=∫p(γ|ε)p(ε|a,b)dε. Since gamma distribution is the conjugate prior of Gaussian distribution [16], a gamma prior is applied to *α*_0_ as:

(21) $$p(\alpha_{0})=\Gamma (\alpha_{0}|q,d),$$

where we set *q,d* → 0 to obtain a broad prior [10,16,23]. Then the prior on ***γ*** is conjugated to the likelihood model given in (19). By using the Bayes’ rule, the joint probability distribution function (PDF) turns into:

(22) $$p(\gamma ,s,\Lambda ,\alpha_{0},\varepsilon )=p(s|\gamma ,\Lambda ,\alpha_{0})p(\gamma |\varepsilon )p(\varepsilon |a,b)p(\alpha_{0})p(\Lambda ),$$

where the off-grid distance model *p*(***Λ***) obeys the uniform distribution, i.e.,:

(23) $$\Lambda ∼U\left({\left[-\frac{1}{2}\varsigma ,\frac{1}{2}\varsigma \right]}^{M}\right).$$

Then the posterior distribution of ***γ***, i.e., $p\left(\gamma |s,\Lambda ,\alpha_{0},\varepsilon \right),$ is a multidimensional Gaussian distribution with mean and covariance matrix as follows [10]:

(24) $$\begin{array}{l} \bar{m}=\alpha_{0}\Sigma \Phi {\left(\Lambda \right)}^{H}s\\ \Sigma ={\left(\alpha_{0}\Phi {\left(\Lambda \right)}^{H}\Phi (\Lambda )+L\right)}^{-1}, \end{array}$$

where ***L*** = *diag*(1/*ε_i_*), *i* = 1,2,…,*M*. To get $\bar{m}$ and Σ, the estimates for *α*_0_, ***Λ*** and ***ε*** are needed. Thus, in the context of Bayesian learning, the problem turns into the search for the hyper-parameters *α*_0_, ***Λ*** and ***ε***.

### 4.3. Bayesian Inference

**Figure 3:**
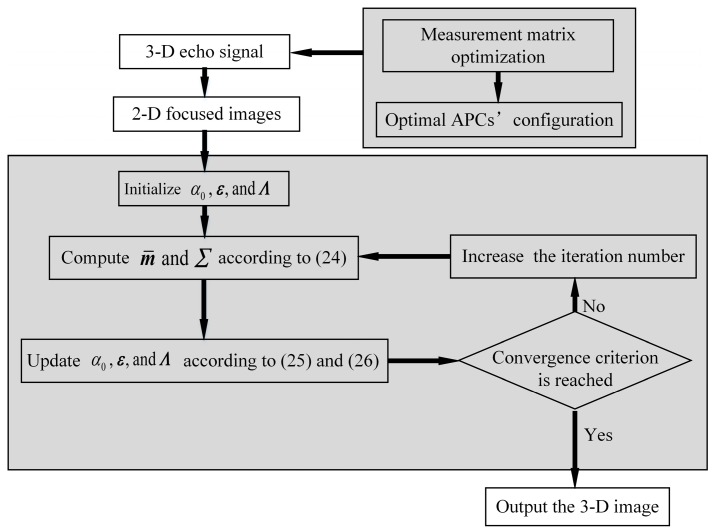
Flowchart of DLSLA 3-D SAR imaging based on measurement matrix optimization and mismatch problem compensation.

Considering $p\left(\Lambda ,\alpha_{0},\varepsilon |s\right)=p\left(s,\Lambda ,\alpha_{0},\varepsilon \right)/p\left(s\right)\propto p\left(s,\Lambda ,\alpha_{0},\varepsilon \right)$, the MAP estimation to estimate *α*_0_, ***Λ*** and ***ε*** is equivalent to maximize the marginal likelihood $p\left(s,\Lambda ,\alpha_{0},\varepsilon \right)$, which can be obtained by integrating the joint PDF given in (22) with respect to ***γ***. By implementing an expectation-maximization (EM) procedure [16], the updates of *α*_0_ and ***ε*** are given by [10]:

(25) $$\begin{array}{cc} \alpha_{0}^{new} & =\frac{N_{e}+q-1}{E\{{\left\|s-\Phi (\Lambda )\gamma \right\|}^{2}\}+d}\\ \varepsilon_{i}^{new} & =\frac{\sqrt{1+4bE\{{\left\|\gamma \right\|}^{2}\}}-1}{2b},\;i=1,2,\cdots ,M, \end{array}$$

where $E\left\{{\left\|\gamma \right\|}^{2}\right\}={\left\|\bar{m}\right\|}^{2}+\Sigma_{ii},\;E\left\{\|s-\Phi \left(\Lambda \right){\gamma \|}^{2}\right\}=\|s-\Phi \left(\Lambda \right){\bar{m}\|}^{2}+a_{0}^{-1}\Sigma_{i=1}^{M}\eta_{i}$, and $\eta_{i}=1-\varepsilon_{1}^{-1}\Sigma_{ii}$ with $\Sigma_{ii}$ being the *i*-th diagonal element of ***Σ***. Then the estimate for ***Λ*** can be achieved by:

(26) $$\Lambda^{new}=\arg\;\underset{\Lambda \in {\left[-\frac{1}{2}\varsigma ,\frac{1}{2}\varsigma \right]}^{M}}{\min}\{\Lambda P\Lambda -2Q^{T}\Lambda \},$$

with:

(27) $$\begin{array}{l} P=R\{{\left(V^{H}V\right)}^{*}⊙(\bar{m}{\bar{m}}^{H}+\Sigma )\}\\ Q=R\{\mathrm{diag}({\bar{m}}^{*})V^{H}(s-R\bar{m})\}-R\{\mathrm{diag}(V^{H}R\Sigma )\}, \end{array}$$

where (●)* denotes the complex conjugate, ***x***⊙***y*** is the element-wise product of ***x*** and ***y***, and R∈(●) takes the real part of a complex variable. As has been seen, hyper-parameters ($\alpha_{0},\Lambda ,\varepsilon )$ and $(\bar{m},\Sigma$) depend on each other, i.e., the aforementioned update equations are obtained by an iterative algorithm. In summary, implement the updates according to Equations (24)–(26) until a convergence criterion is satisfied, then the posterior distribution function for ***γ*** and the estimate for ***Λ*** are obtained, respectively. By combining the measurement matrix optimization and the mismatch compensation, the flowchart of the proposed DLSLA 3-D SAR imaging algorithm is presented in Figure 3.

## 5. Experiments and Results

In this section, we carry out simulated experiments to verify our proposed algorithms. All experiments are performed on a PC with a 3.4 GHz Intel i7 CPU and the Windows 7 system (MathWorks Inc., Natick, MA, USA). The system parameters of DLSLA 3-D SAR are listed in Table 1.

### 5.1. Performance Comparison of Measurement Matrix Optimization Schemes

**Figure 4:**
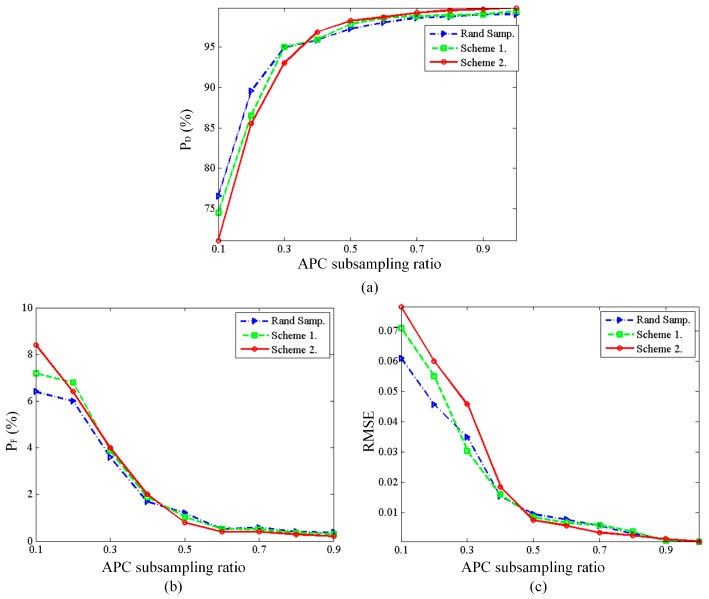
Reconstruction performance by different APCs’ configurations schemes: (**a**) probability of detection vs. APC subsampling ratio; (**b**) probability of false alarm vs. APC subsampling ratio; (**c**) RMSE vs. APC subsampling ratio.

In this subsection, we present the simulated results of cross-track reconstruction by different APCs’ configurations based on the proposed sampling strategies. According to the system parameters we know that the cross-track Rayleigh resolution [5] *ρ_c_* = *λR*(*m,n*;*P*)/(2*L_c_*) is about 1.6 m, and the cross-track reconstruction pixel grid is chosen to match the Rayleigh resolution. Considering the spatial sparsity of a 3-D scene, we assume that ten scatterers, which have the same wave-propagation and along-track locations, randomly distribute on different cross-track pixel grids in the effective imaging region [3]. The on-grid scatterers are all with unit magnitudes and uniform random phases in the range (–π,π). The sparse recovery method basis pursuit denoise (BPDN) [24] is used for the reconstruction. Over 100 Monte Carlo simulations, the reconstruction performance is evaluated by the probability of detection (*P_D_*), probability of false alarm (*P_F_*) and relative mean square error (RMSE). RMSE defined as RMSE = ${\left\|\hat{\gamma }-\gamma \right\|}_{2}^{2}/{\left\|\gamma \right\|}_{2}^{2}$ is used to evaluate the relative error between the estimated value $\hat{\gamma }$ and the true reflectivity coefficient vector γ. By defining *H*_1_ and *H*_0_ as the event of presence of the scatterer at the given grid and the event of absence of the scatterer, respectively, *P_D_* and *P_F_* are defined as:

(28) $$\begin{array}{l} P_{D}=\mathrm{Pr}\{|{\hat{\gamma }}_{\overset{⌢}{x}}|\geq \partial |H_{1}\}\\ P_{F}=\mathrm{Pr}\{|{\hat{\gamma }}_{{\overset{⌢}{x}}_{C}}|\geq \partial |H_{0}\} \end{array}$$

where $\hat{x}$ is the set of the grids on which the true scatterers are located, ${\hat{x}}_{c}$ is the complement of $\hat{x}$, ∂ is the threshold and is set to 0.4 in this scene. With SNR = 20 dB, random sampling strategy is carried out to compare with the two proposed schemes, and Figure 4 presents the *P_D_*, *P_F_* and RMSE versus different APC subsampling ratios (defined as the value of *N_e_*/*M*). As can be seen, when the number of APCs is very low, all the APCs’ configuration schemes cannot give good reconstruction performance. Notice that when the APC subsampling ratio is low, the configuration scheme based on the worst-case mutual coherence performs better than the scheme based on the modified average mutual coherence. While when the APC subsampling ratio increases to 0.4, the latter scheme gives a better performance and can reconstruct the scatterers with a detection rate higher than 95%, false alarm rate lower than 2%, and reconstruction error lower than 0.02. It can be seen that the random sampling scheme and the worst-case based scheme have a small gap at most APC subsampling ratios. The evaluated values may vary with the system parameters, while the statistical trend is evident and can at least provide a reference for practical application.

### 5.2. Reconstruction Performance for Off-Grid Scatterers

In this subsection, we consider five scatterers with the same wave-propagation and along-track locations, but different cross-track locations to compare the cross-track reconstruction performance. The cross-track reconstruction grid is equal to the Rayleigh resolution. The first and third scatterers are on the grids, while the other three scatterers are deviated from the grids. The parameters of OGSBI are set as *a* = 1, *b* = 10^–2^ and *q* = *d* = 10^–4^. The initializations are chosen as $\Lambda^{\left(0\right)}=0,\alpha_{0}^{\left(0\right)}=100/Var\left\{s\right\}$, and $\varepsilon^{\left(0\right)}=\left|R^{H}s\right|$, where *Var*{·} denotes the variance. We compare OGSBI with BPDN and orthogonal matching pursuit (OMP) [25] on both reconstruction performance and the computational time.

**Figure 5:**
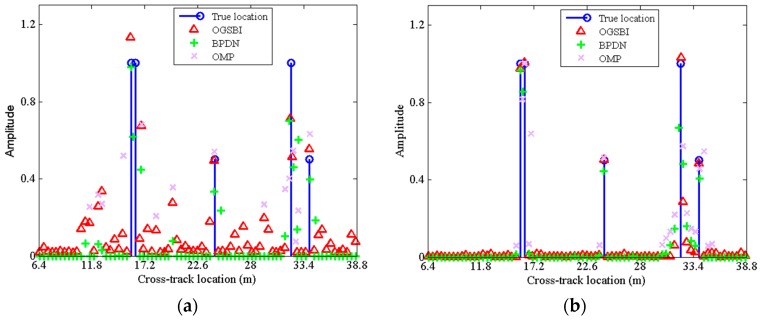
Cross-track 1-D reconstructed profiles by three methods with (**a**) SNR = 5 dB; (**b**) SNR = 25 dB.

Figure 5 gives the cross-track 1-D profiles of the reconstructed results with the APC subsampling ratio 0.5, and two noise levels, SNR = 5 dB and 25 dB, respectively. As shown in Figure 5a, when the SNR is low, all the methods have spurious estimations since the measurement noise dominates the uncertainties. Even though, we can see that the locations of the reconstructed peaks by OGBSI still approximate the true values. When the SNR is high enough as shown in Figure 5b, the OGBSI method adopted in this paper can reconstruct both the off-grid and on-grid scatterers accurately. BPND can reconstruct the on-grid scatterer but suffer from many spurious estimations for those off-grid scatterers. Compared with BPDN, OMP also fails to reconstruct the off-grid scatterers accurately, and tends to miss the closely separated scatterers or the weak scatterer.

Considering the computation load, we recall that the number of measurements, the number of imaging grids, and the number of non-zero scatterers are *N_e_*, *M* and *N_p_*, respectively, and the relation between them is *N_p_* < *N_e_* < *M*. The computational complexity of BPDN is *O*(*M*^3^) per iteration, while the complexity of OMP is about *O*(*MN_p_N_e_*). For the implementation of OGSBL, the main computational burden lies in updating Equation (24), thus, the OGSBI has a computational complexity of *O*$\left(N_{e}^{2}M\right)$ per iteration. In the above experiments, OGSBI, BPDN and OMP take about 0.16, 0.78, and 0.0057 s, respectively.

**Figure 6:**
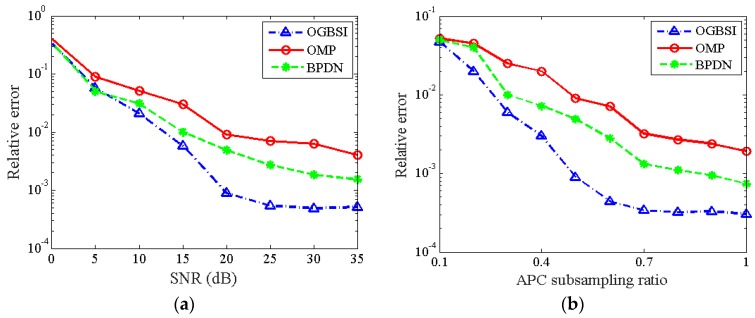
Reconstruction performance for off-grid scatterers: (**a**) relative error of cross-track reconstruction vs. SNR; (**b**) relative error of cross-track reconstruction vs. APC subsampling ratio.

To quantify the reconstruction performance by the three methods, Figure 6 shows the relative error of cross-track reconstruction with respect to various APC subsampling ratios and SNRs. In the sequel, the relative error of cross-track reconstruction is defined as $\frac{1}{N_{p}}\Sigma_{i}^{N_{p}}\|{\tilde{x}}_{i}-x_{i}{\|}_{2}/{\left\|x_{i}\right\|}_{2}$, presenting the relative error of cross-track locations between the reconstructed and ground true value. Since the reconstruction may suffer from spurious scatterers, the largest five scatterers are chosen as the estimates for different methods. For different APC subsampling ratios, the APCs’ configuration scheme is chosen as the one with the lowest RMSE in the case of on-grid scatterers reconstruction among the afore-mentioned three schemes. Figure 6a presents the results with respect to different SNRs with APC subsampling ratio *N_e_*/*M* = 0.5. As can be seen, all the methods have large errors in the low SNR region, where the noise affects the performance dominatingly. As the SNR increases, the OGSBI outperforms the BPDN and OMP, which suffer from an error low bound caused by the deviation between the true locations and the nearest grids. Similarly, Figure 6b gives the relative error with SNR = 25 dB and various APC subsampling ratios. As can be seen from the statistical results, the performance of the cross-track reconstruction algorithm based on OGBSI is stable and robust at moderate levels of SNRs and APC subsampling ratios. Moreover, the experiments show that the sparse Bayesian inference based method can reconstruct the off-grid scatterers accurately with a coarse grid scheme.

Known from the above experiments, we can see that the OGSBI algorithm can provide accurate cross-track reconstruction for off-grid scatterers, with a moderate level of SNR and APC subsampling ratios. It does not need the regularization parameter or a denser sampling grid. Its computational complexity is between that of BPDN and OMP, which both suffer from off-grid effect. Particularly, with coarse imaging grids, the reconstruction performance and computational efficiency are acceptable for 3-D SAR image cross-track reconstruction.

### 5.3. Experiment for Distributed 3-D Imaging Scene

**Figure 7:**
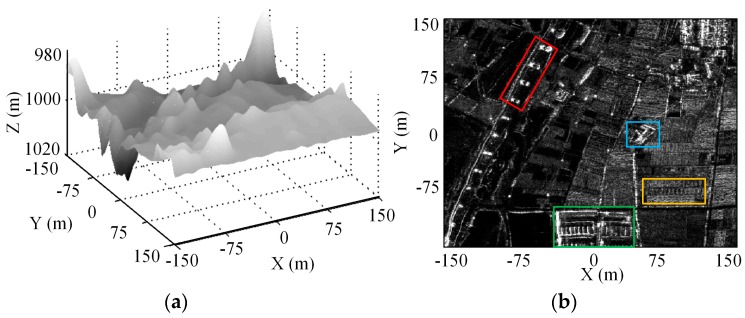
3-D imaging scene simulation. (**a**) Airborne DEM; (**b**) 2-D circular SAR image.

**Figure 8:**
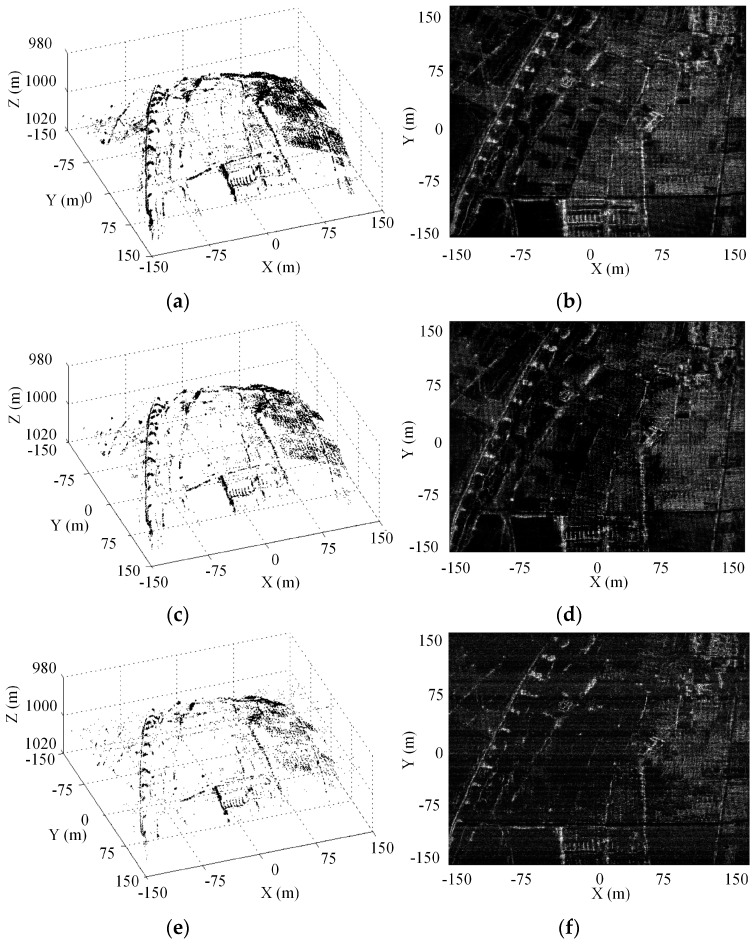
3-D reconstructed image in Cartesian coordinate system: (**a**) OGSBI; (**c**) BPDN; (**e**) OMP. *XOY* plane orthographic projection image: (**b**) OGSBI; (**d**) BPDN; (**f**) OMP.

In this subsection, we use a simulated 3-D distributed imaging scene to show the reconstruction performance. The scatterers distribute uniformly in the along-track dimension in a region of [−150 m, 150 m] with 1.5 m interval, while with the same region but ±20% random deviations from the cross-track grids to simulate the off-grid. As shown in Figure 7, the elevation locations and reflectivity coefficients come from airborne DEM data and a 2-D circular SAR (CSAR) image [26], respectively. 60% APCs are chosen from the filled array according to the optimal APCs’ configuration scheme. Gaussian noise with SNR 20 dB is added to the echo signal. Figure 8a,c,e show the 3-D reconstructed images of which the cross-track reconstruction are obtained by OGSBI, BPDN and OMP respectively. The orthogonal projection images onto the *XOY* plane by the three methods are presented in Figure 8b,d,f, respectively. As can be seen, BPDN and OMP, without compensating the off-grid mismatch problem, both suffer from many spurious estimated scatterers and loses many image details. Compared with the original simulated input image, the reconstructed images of BPDN and OMP seem sparser, which indicates that many features have been discarded. In contrast, after compensating the mismatch problem, OGSBI provides reconstructed image with better contrast and more details.

**Figure 9:**
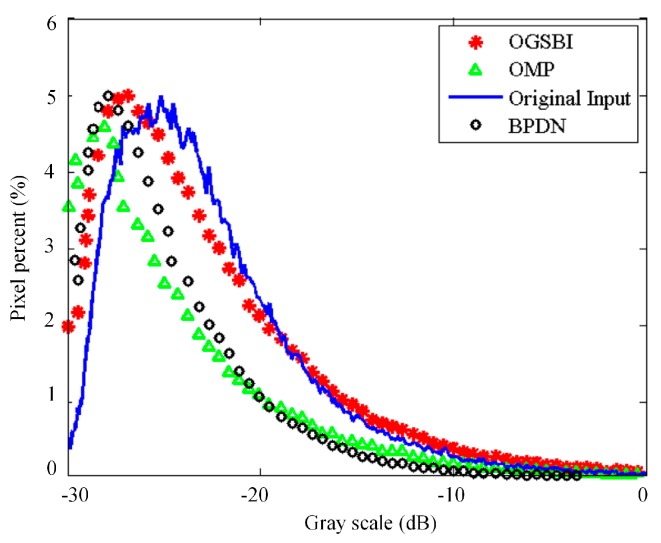
Grayscale histogram comparison of the input 2-D image and the orthographic projection reconstructed images by the three sparse recovery methods.

Figure 9 gives the grayscale histograms for the input 2-D image and the orthographic projection reconstructed images by the three sparse recovery methods. As can be seen, the histogram of the orthographic projection reconstructed image by OGSBI is close to that of the original input image. The histograms for BPDN and OMP both have offset compared with that of the OGSBI, and OMP produces the histogram with a smaller dynamic range. This phenomenon also indicates that the orthographic projection reconstructed images obtained by BPDN and OMP lose image details. Finally, we use four targets regions that marked in Figure 7b to quantify the reconstruction performance. To evaluate the error between the reconstructed and the ground true values, Table 2 shows the relative error of cross-track reconstruction. Compared with the other two methods, OGSBI can obtain a smaller reconstruction error, especially for the regions with weak reflectivity coefficients, such as the region marked by the orange rectangle in Figure 7b.

## 6. Conclusions

This paper focuses on two major problems that affect the properties of the measurement matrix when applying compressive sensing (CS)-based methods for downward looking sparse linear array three-dimensional synthetic aperture radar (DLSLA 3-D SAR) cross-track reconstruction. Two mutual coherence-based criteria are proposed to optimize the configuration of antenna phase centers (APC), which determines the form of the measurement matrix. On the one hand, based on the worst-case mutual coherence, the cyclic difference sets or the modified Lloyd numerical search method can be used to obtain the optimized measurement matrix in a tight frame. On the other hand, the modified average mutual coherence is used to optimize the measurement matrix, which can give a good average reconstruction performance. Experiments for DLSLA 3-D SAR cross-track reconstruction test the performance of the proposed APCs’ configuration schemes with respect to detection rate, false alarm rate and reconstruction error. In the meantime, the mismatch problem of measurement matrix caused by the off-grid scatterers is considered in this paper, and the off-grid sparse Bayesian inference (OGSBI) method is applied to solve this problem. Simulated experiments show the superior performance of OGSBI compared to those CS based methods which do not consider the off-grid effect.

## Figures and Tables

**Table 1:** Simulation parameters.

Parameter	Value	Parameter	Value	Parameter	Value
Center Wavelength	8 mm	Frequency Points	1600	AT ^2^ Sampling Number	261
Signal Bandwidth	300 MHz	Platform Flying Height	1000 m	AT Sampling Interval	0.01 m
A/D Sampling Frequency	360 MHz	CT ^1^ APC Number	261	CT Beam Width	14°
Signal Pulse Width	4 μs	CT Sampling Interval	0.01 m	AT Beam Width	14°

^1^ CT is short for cross-track; ^2^ AT is short for along-track.

**Table 2:** Relative error of cross-track reconstruction.

	Region I	Region II	Region III	Region IV
OGSBI	0.21	0.33	0.24	0.41
BPDN	0.26	0.42	0.32	0.56
OMP	0.33	0.47	0.51	0.72

Regions I~IV stand for the regions marked by red, green, blue, and orange rectangles in Figure 7b.

## Abbreviations

DLSLA 3-D SAR	Downward looking sparse linear array three-dimensional synthetic aperture radar
CS	Compressive sensing
APC	Antenna phase centers
RIP	Restricted isometry property
CDS	Cyclic difference sets
OGSBI	Off-grid sparse Bayesian inference
MAP	Maximum a posteriori
MIMO	Multiple-input multiple-out
SVQ	Sphere vector quantization
PDF	Probability distribution function
EM	Expectation-maximization
BPDN	Basis pursuit denoise
RMSE	Relative mean square error
OMP	Orthogonal matching pursuit
CSAR	Circular SAR
